# Supplementary material for: Structural basis for differential inhibition of eukaryotic ribosomes by tigecycline
Source: Nat Commun. 2024 Jun 28;15:5481. doi: 10.1038/s41467-024-49797-7 (PMC11213857; doi:10.1038/s41467-024-49797-7)
Supplement: Supplementary file 1 — Supplementary Information [file 41467_2024_49797_MOESM1_ESM.pdf]

Structural basis for differential inhibition of eukaryotic ribosomes by tigecycline

Xiang Li[1,3], Mengjiao Wang[1,3], Timo Denk[2,3], Robert Buschauer[2], Yi Li[1], Roland Beckmann[2*], and Jingdong Cheng[1*]

[1] Minhang Hospital & Institutes of Biomedical Sciences, Shanghai Key Laboratory of Medical Epigenetics, International Co-laboratory of Medical Epigenetics and Metabolism, Fudan University, Dong'an Road 131, 200032, Shanghai, China

[2] Gene Center, Ludwig-Maximilians-Universität München, Feodor-Lynen-Straße 25, 81377 Munich, Germany

[3] These authors contributed equally: Xiang Li, Mengjiao Wang, Timo Denk.

*Correspondence: beckmann@genzentrum.lmu.de (R.Be.), and cheng@fudan.edu.cn (J.C.)

a

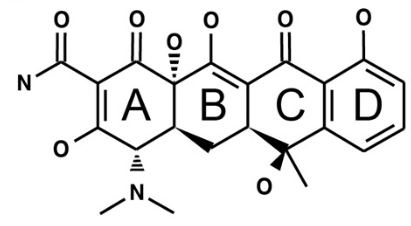

Tetracycline (Tet)

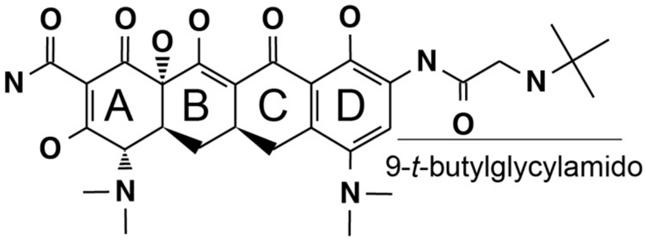

Tigecycline (Tig)

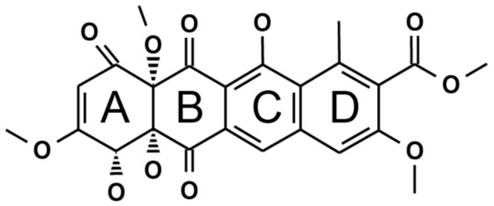

Tetracenomycin X (TcmX)

Supplementary Figure 1. Chemical structures of the Tetracycline, Tigecycline and Tetracenomycin X.

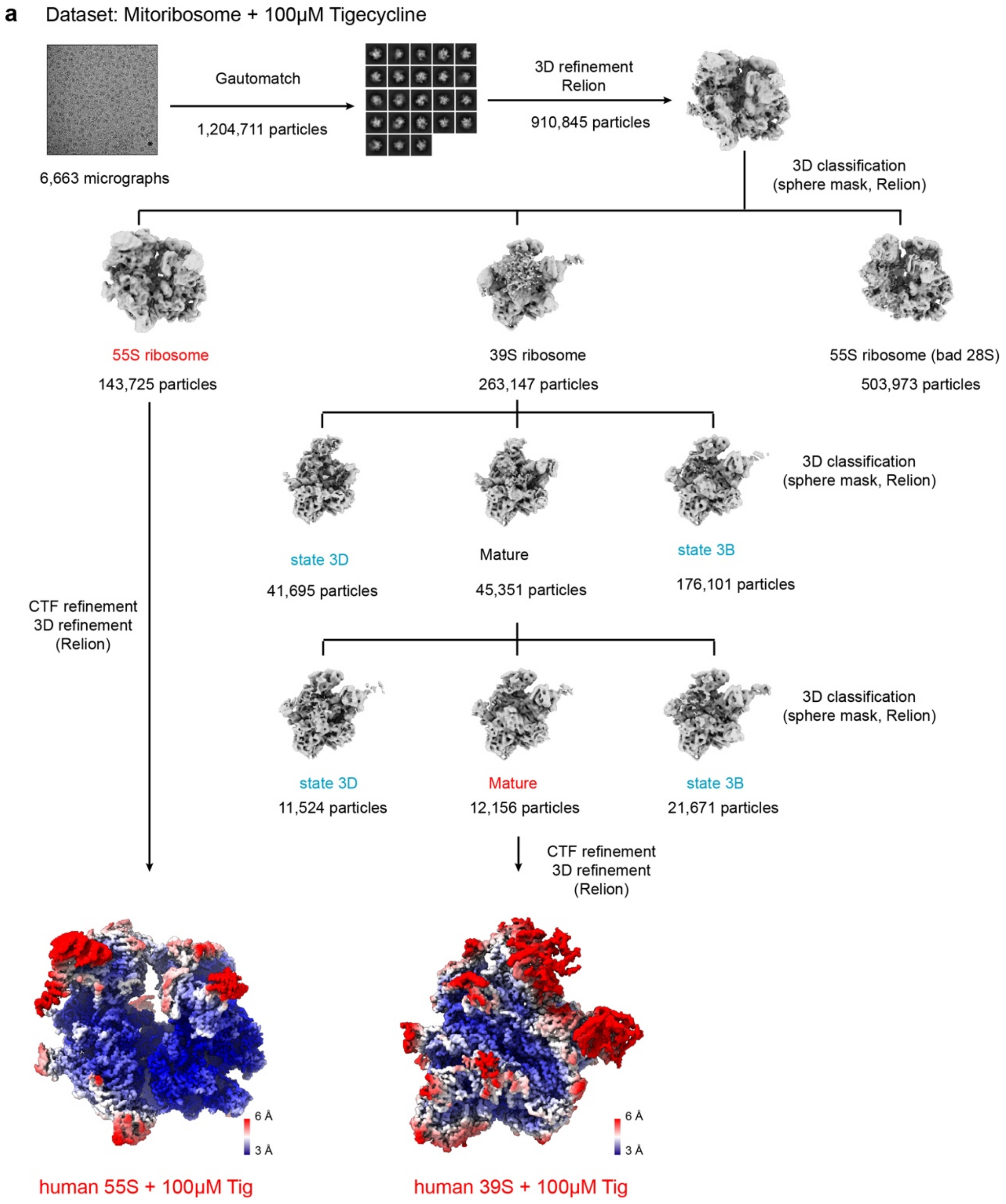

Supplementary Figure 2. The sorting scheme of the human mitoribosome with 100 µM tigecycline dataset. a, Finally, the classes representing mature 55S and 39S mitoribosomes were selected and refined to high resolution using Relion with 55S and 39S mitoribosome masks, respectively. The procedure, masks and software used are listed alongside. The published states of the immature 39S mitoribosome are labeled in cyan.

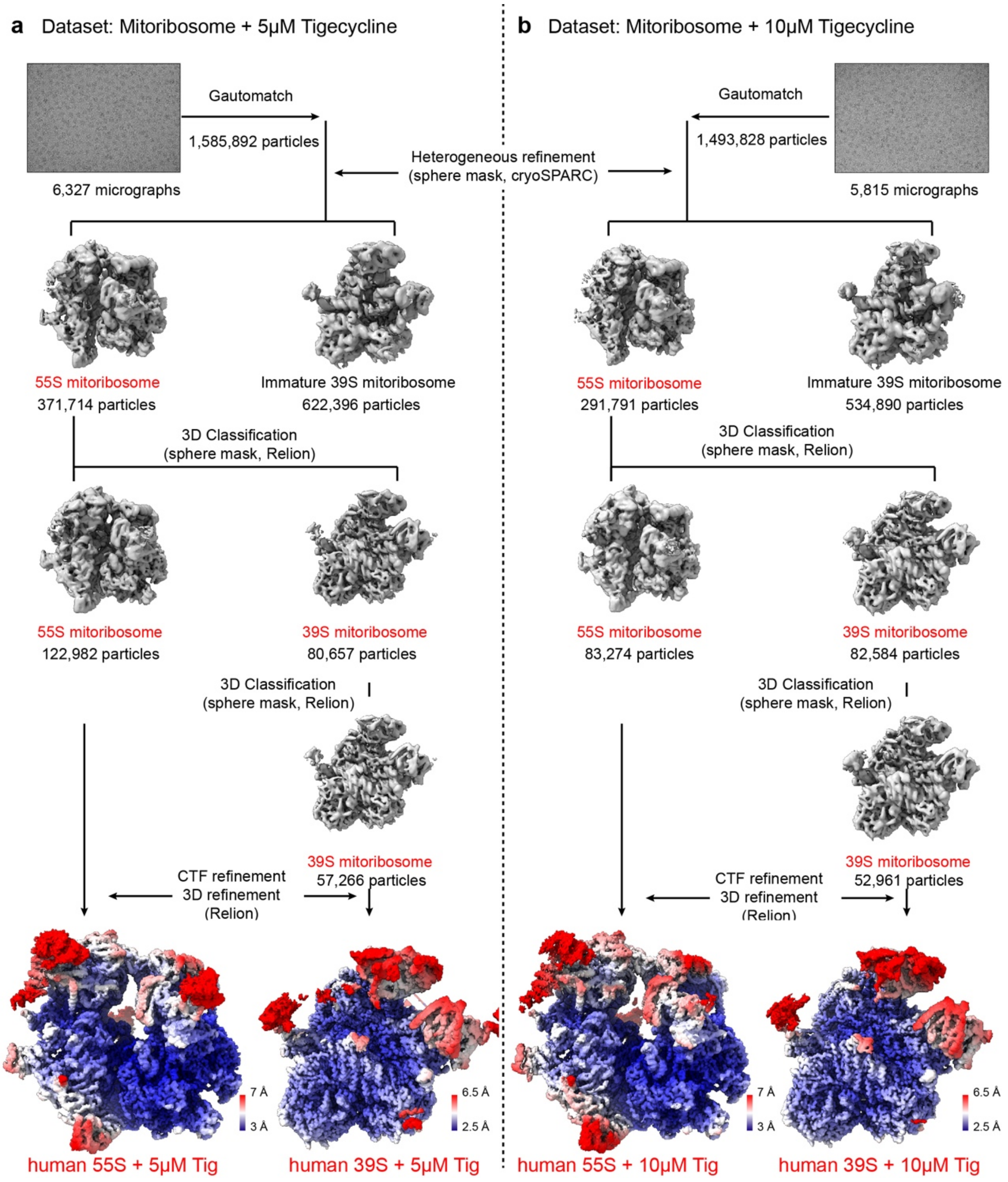

Supplementary Figure 3. The sorting schemes of the human mitoribosome with 5 μM and 10 μM tigecycline datasets. a-b, Finally, for both with 5μM (**a**) and 10μM (**b**) tigecycline, the classes representing mature 55S and 39S mitoribosomes were selected and refined to high resolution using Relion with 55S and 39S mitoribosome masks, respectively. The procedure, masks and software used are listed alongside.

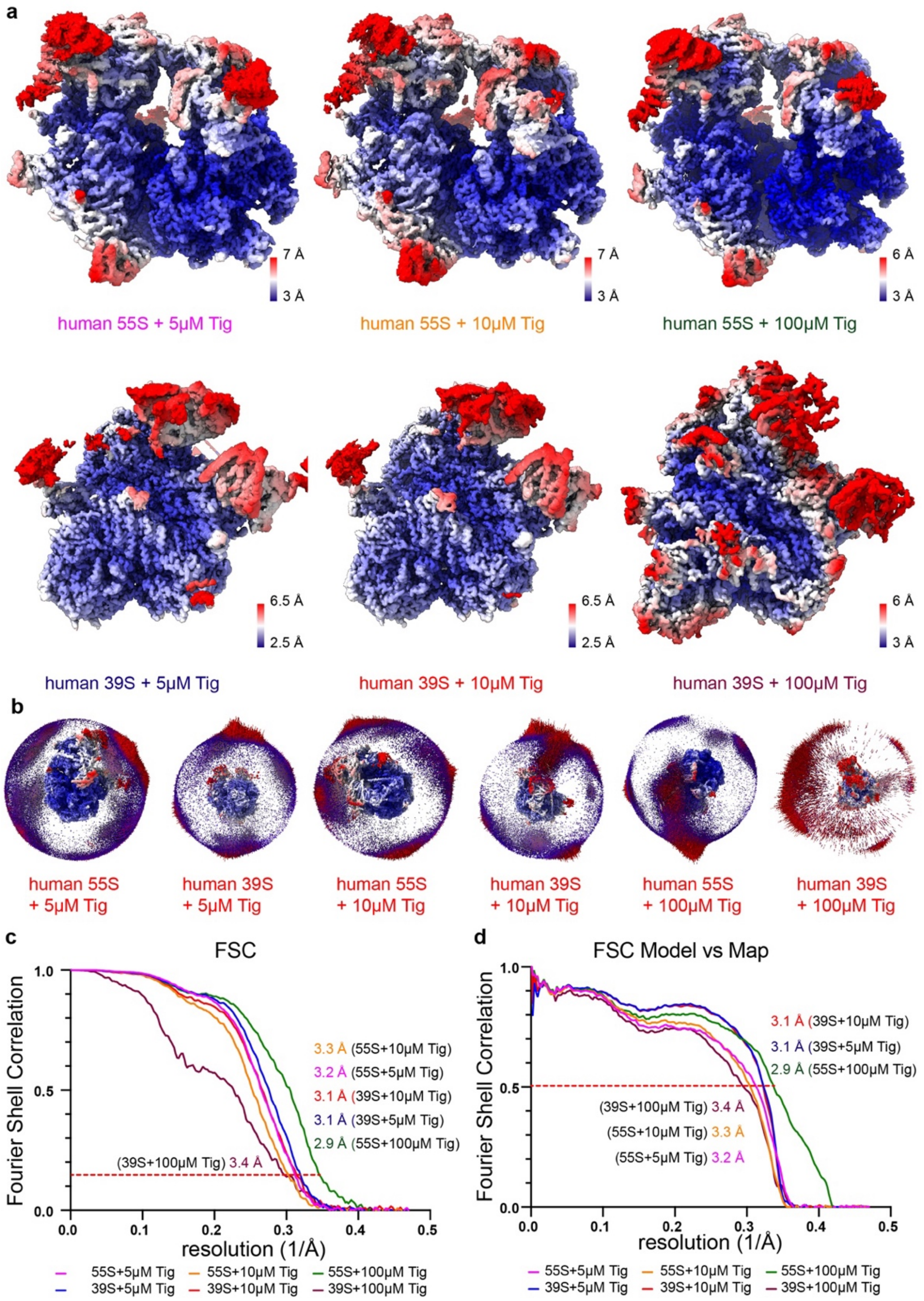

Supplementary Figure 4. The local resolution distribution and FSC curves of the human mitoribosomes with tigecycline. a-b, Cryo-EM maps of human 55S (**top**) and 39S (**bottom**) ribosomes with 5μM, 10μM and 100μM tigecycline were filtered according to their local resolution estimation. The scale bars are shown alongside (**a**), accompanied by the Euler angle distribution of particles involved in the final reconstructions with larger red cylinders denoting orientations encompassing a higher particle number (**b**). **c-d,** The color-coding corresponds to Fourier Shell Correlation (FSC) curves of the maps (**c**) and the model-to-map correlation curves (**d**) were calculated in Relion. The corresponding resolution was estimated using either the 0.143 or 0.5 cutoff criterion (red dotted line).

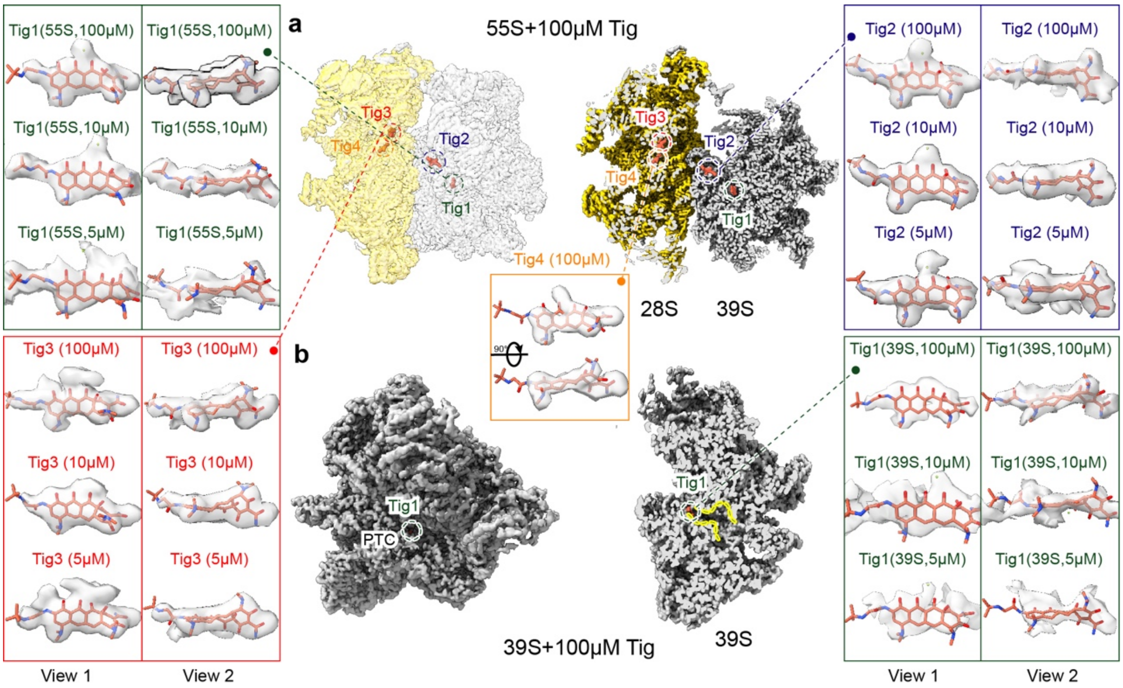

Supplementary Figure 5. Cryo-EM structures of tigecycline on the human mitoribosome. a-b, Overview (left panel) and cross section (right panel) of the cryo-EM maps of the 55S (**a**, 28S, yellow; 39S, gray) and 39S mitoribosome (**b**, gray) showing four tigecycline (at various concentration levels, tomato red) binding sites within the large and small subunits. The Tig1-3 binding sites were consistent across all concentrations, except for Tig4, which only appeared under the 100μM tigecycline condition. Two different views of the tigecycline density are shown in their corresponding boxes. The overview of the 55S mitoribosome density is shown in translucent, while the densities of the tigecycline molecules are not. Densities for Tig2-4 are derived from the cryo-EM density map post-processed by DeepEMhancer, whereas the density of Tig1 were derived from the consensus map without post-processing.

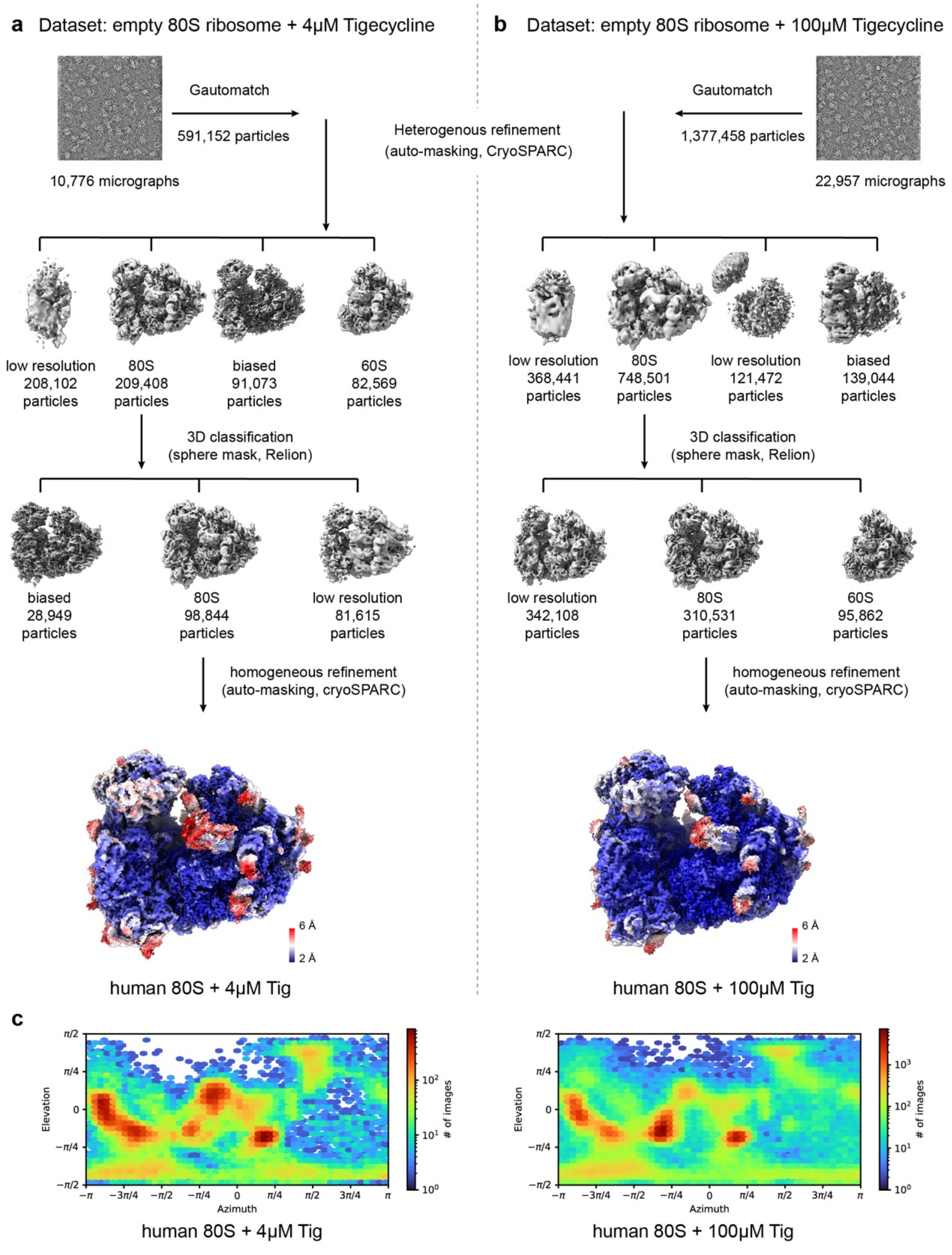

Supplementary Figure 6. The sorting scheme of the empty human 80S ribosome with tigecycline dataset. a-b, the detailed sorting schemes of the empty 80S ribosome incubated with 4 μM (**a**) or 100 μM (**b**) tigecycline are shown. Finally, the 80S ribosome class was picked and refined to high resolution using cryoSPARC with auto-masking. The procedure, masks and software used are listed alongside. **c,** The corresponding particle angular distribution heatmaps generated by cryoSPARC are shown at the bottom.

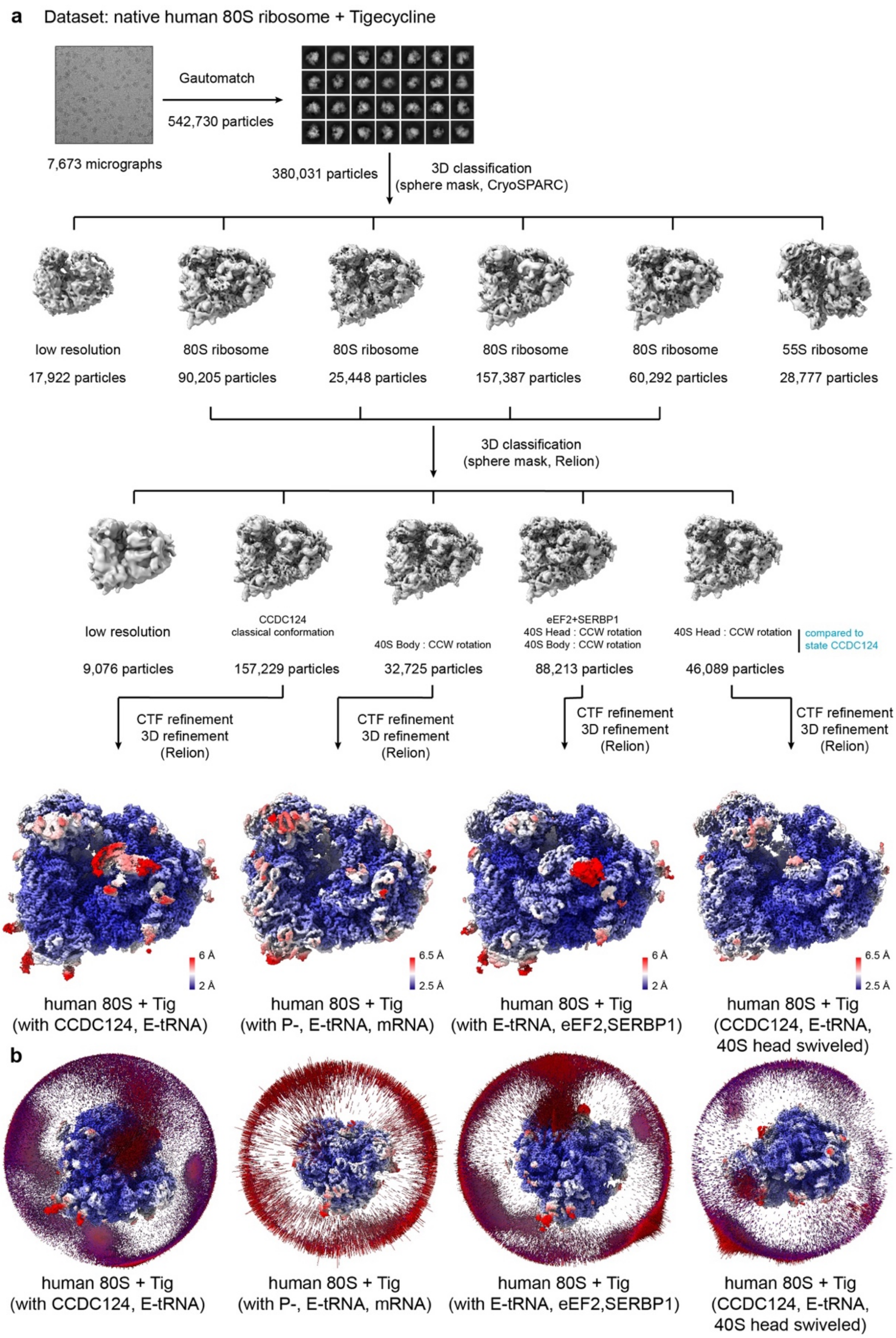

Supplementary Figure 7. The sorting scheme of the human 80S ribosome with tigecycline dataset. a-b, Finally, the four classes of 80S ribosome which are at high resolution were picked and refined to high resolution using Relion with 80S ribosome mask (a), their respective Euler angle distributions are shown at the bottom, where each bar represents the particle count, and the color intensity transitions from blue to red, indicating increasing frequency and corresponding to different orientations (b). The procedure, masks and software used are listed alongside.

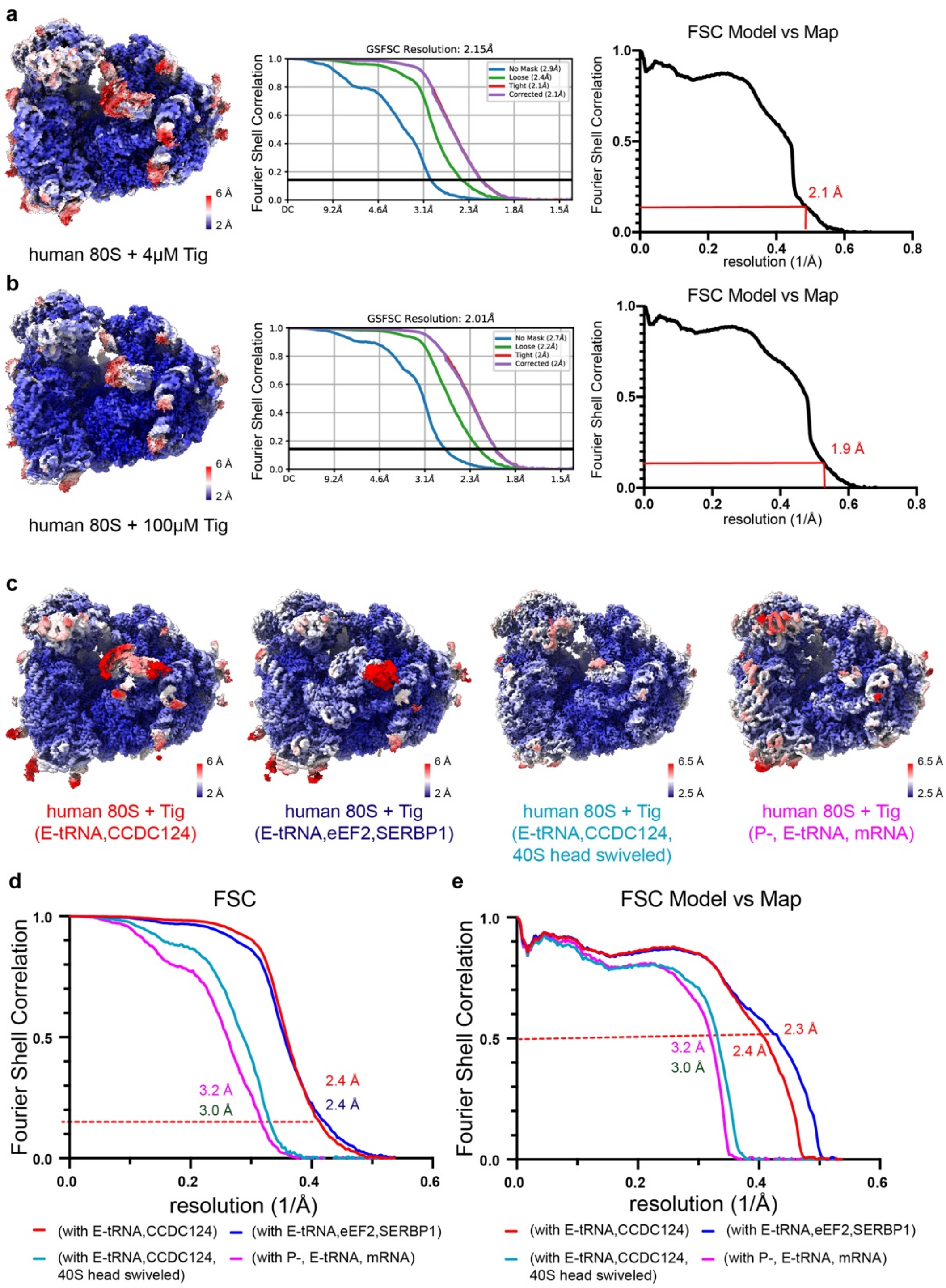

Supplementary Figure 8. The local resolution distribution and FSC curves of the human 80S ribosomes. a-b, the cryo-EM maps of the empty 80S ribosome, incubated with 4 μM (**a**) or 100 μM (**b**) tigecycline, were filtered according to their local resolution estimation in cryoSPARC. Their corresponding FSC curves are shown in the middle, while the model-to-map correlation curves are shown on the right. **c,** Cryo-EM maps of human 80S ribosomes from four different states were filtered according to their local resolution estimation. **d-e,** The color-coded corresponding Fourier Shell Correlation (FSC) curves of the maps (**d**) and the model-to-map correlation curves (**e**) were calculated in Relion. The corresponding resolution was estimated using either the 0.143 or 0.5 cutoff criterion (red dotted line), respectively.

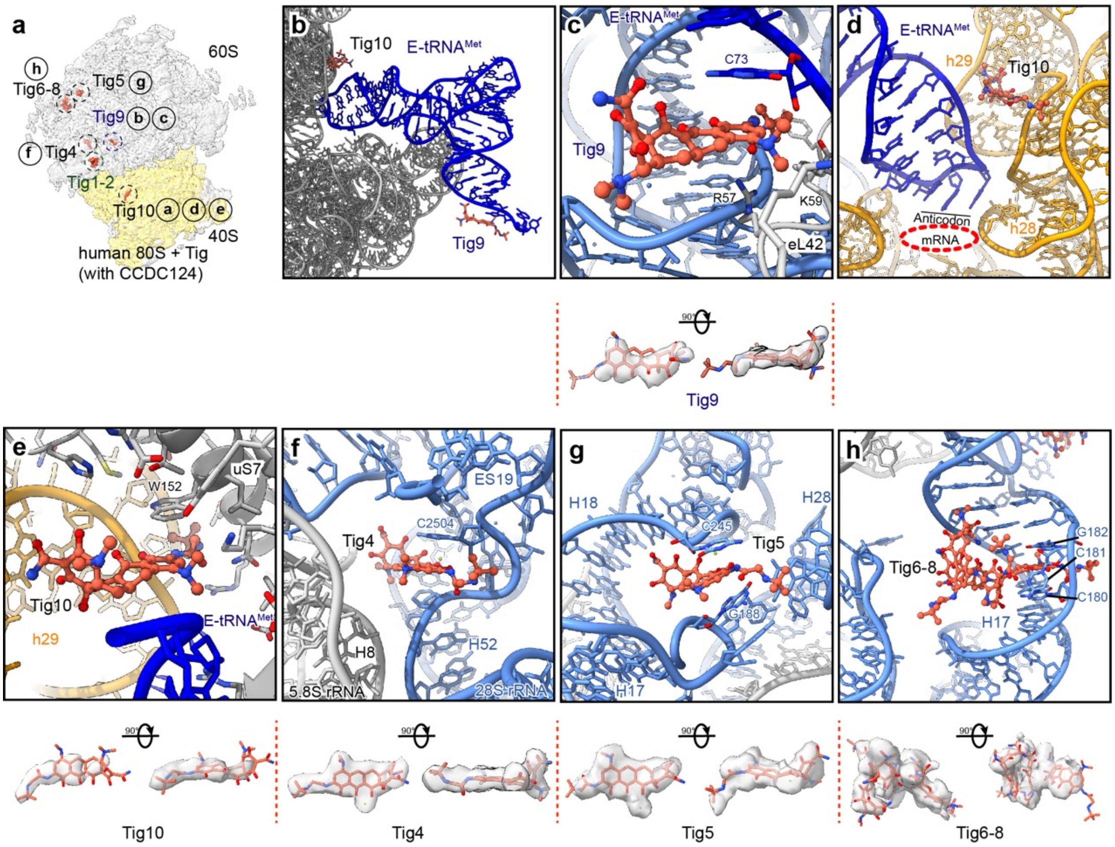

Supplementary Figure 9. Human 80S ribosome specific Tigecycline binding sites. a, Overview of the tigecycline binding sites on the human 80S ribosome (human 80S+tig (with CCDC124) state). **b**, A zoom-in view of the binding sites for Tig9 and Tig10 molecules around the E-tRNA^{Met}. **c**, The Tig9 molecule stacks in between base C73 of the E-tRNA^{Met} and residue R57 of the eL42. **d-e**, Tig10 is trapped in h29 of the18S rRNA (**d**) with its 9-*t*-butylglycylamido moiety buried inside of a hydrophobic pocket formed by uS7 (**e**), and its tetra ring is close to the E-tRNA^{Met}. **f**, Tig4 molecule interacts with H52 of the with its D-ring tightly stacking with base C2504. **g**, Tig5 molecule stacks in between bases C245 and G188 of the 28S rRNA helix 18. **h**, Tig6-8 molecules interact with 28S rRNA Helix 17. Two different views of the corresponding Tigecycline density are displayed as below.

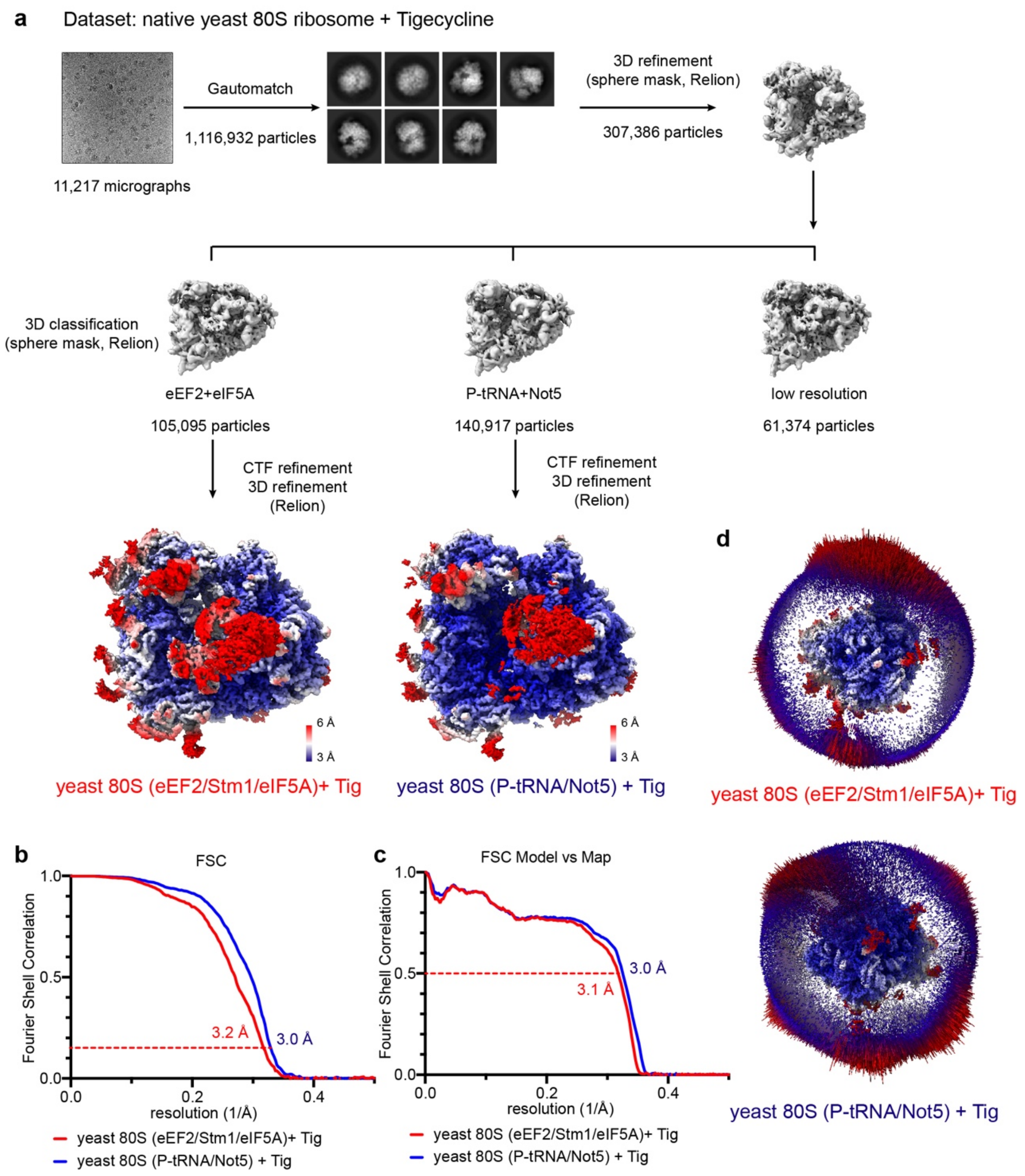

Supplementary Figure 10. The data processing of the yeast 80S ribosome with tigecycline dataset. a, the sorting scheme of the dataset collected from the yeast 80S ribosome treated with tigecycline. The selected classes are refined to high resolution using Relion with 80S ribosome mask. The procedure, masks and software used are listed alongside. **b-c,** The corresponding Fourier Shell Correlation (FSC) curves of the map (**b**) and the model-to-map correlation curves (**c**) for the yeast 80S ribosome were calculated in Relion. The corresponding resolution was estimated using either the 0.143 or 0.5 cutoff criterion (red dotted line), respectively. **d,** The corresponding Euler angle distributions of the maps were generated using Relion.

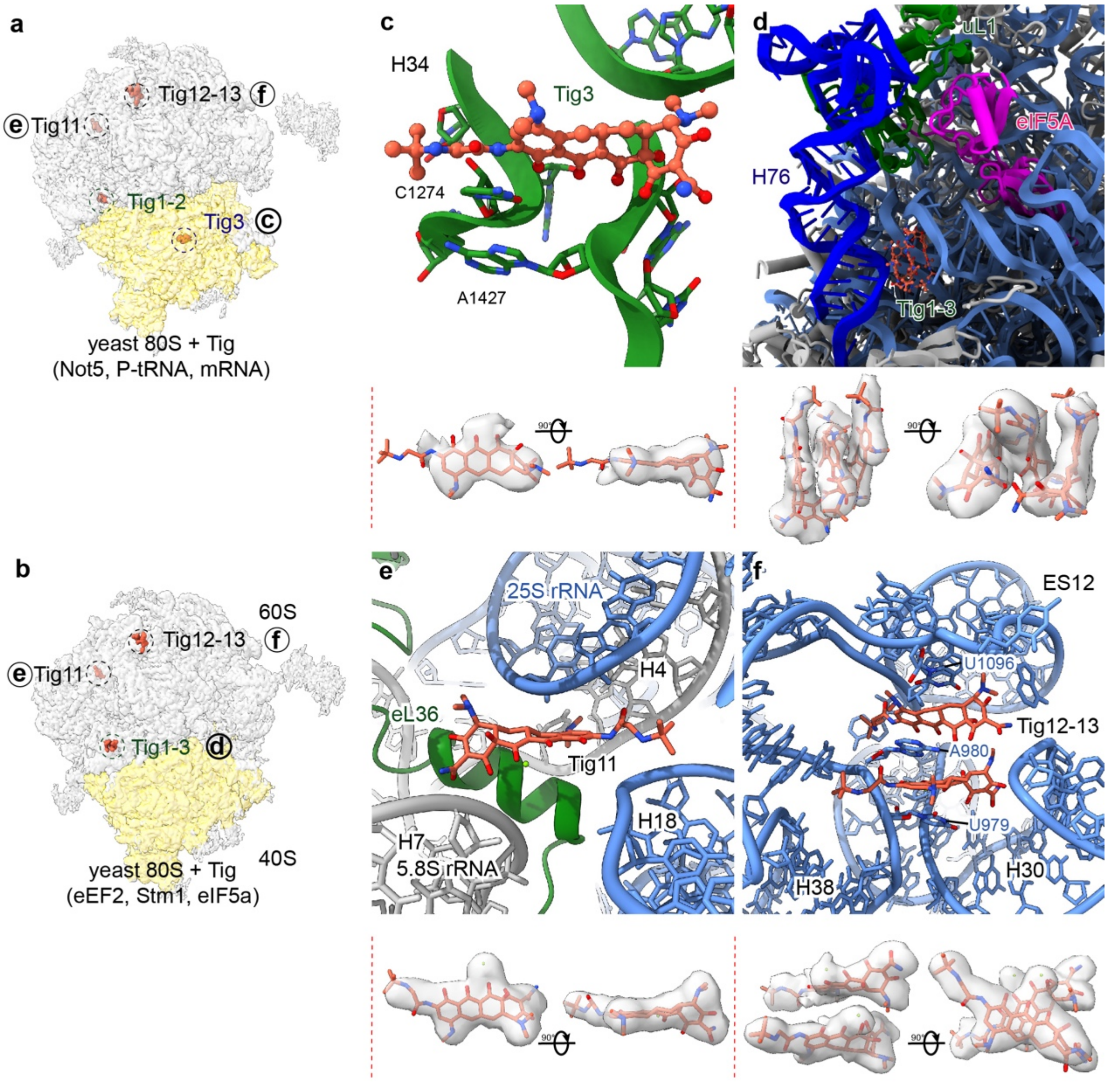

Supplementary Figure 11. Yeast 80S ribosome specific Tigecycline binding sites. a-b, Overview of the tigecycline binding sites in states yeast 80S+Tig (with Not5/P-tRNA/mRNA) and yeast 80S+Tig (with eEF2/Stm1/eIF5a). **c**, Tig3 molecule binds to the universal conserved "primary" binding site on h34 of the 18S rRNA. **d**, Detailed view of the Tig1-3 molecules from the yeast 80S (eEF2/Stm1/eIF5A) structure. The density map is original from the consensus map filtered with DeepEMhancer. Like the Tig1-2 molecules from the yeast 80S ribosome (Not5/P-tRNA/mRNA), the Tig1-3 molecules also bind near the L1 stalk. **e**, The Tig11 molecule interacts with eL36 and 25S rRNA (Helix 18) and 5.8S rRNA (Helix 4, Helix 7). **f,** Tig12-13 molecules interact with H30, H38 and ES12 of the 25S rRNA. They stack in between bases U979, A980 and U1096. Two different views of the corresponding Tigecycline density are displayed as below.

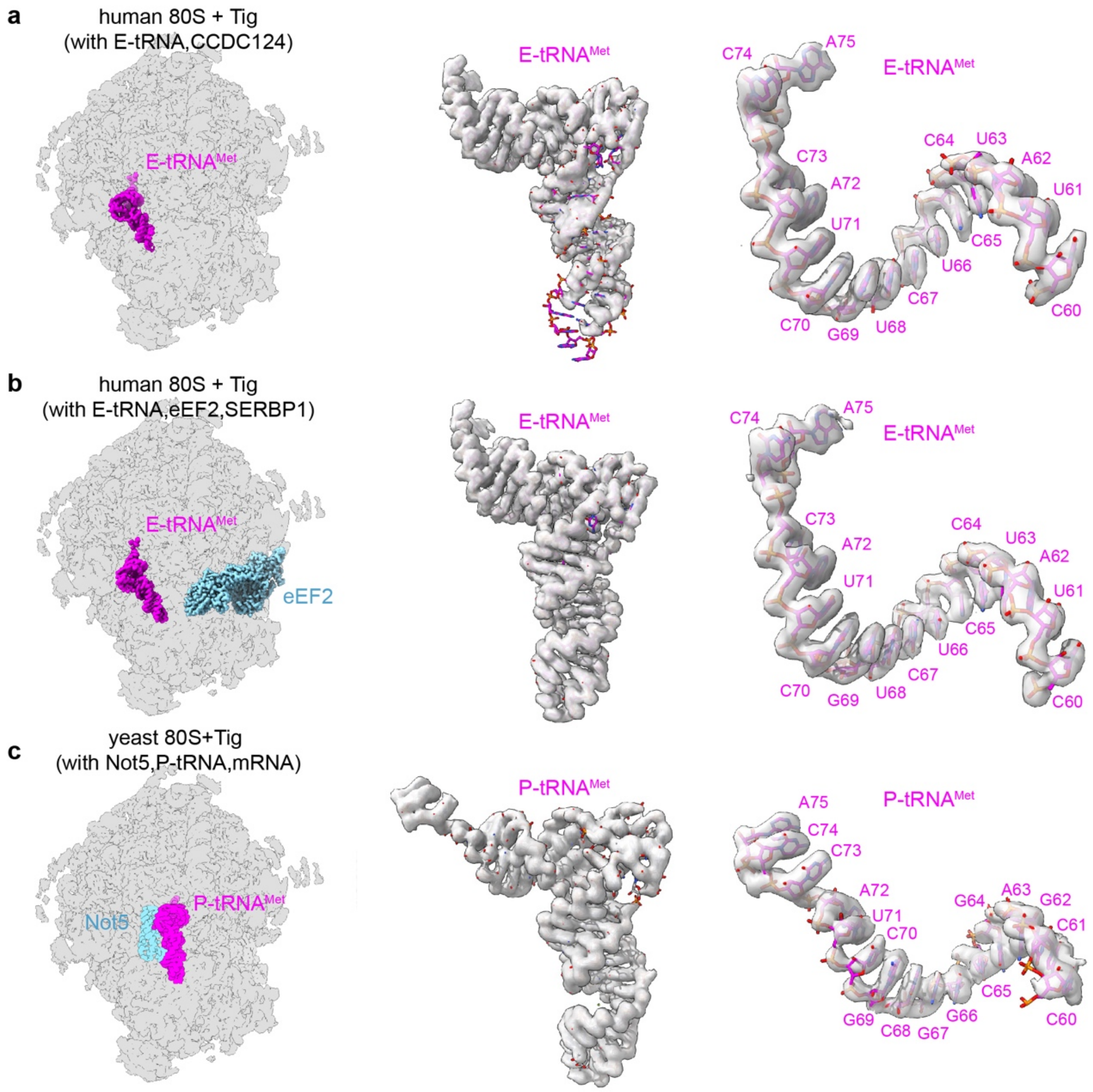

Supplementary Figure 12. Human 80S ribosome specific Tigecycline binding sites. a-c, Three cryo-EM maps of human or yeast 80S ribosome (in our sample) containing tRNA inside were analyzed, including human 80S+Tig (with E-tRNA, CCDC124) (**a**), human 80S+Tig (with E-tRNA, eEF2, SERBP1) (**b**), and yeast 80S+Tig (with Not5, P-tRNA, mRNA) (**c**). The overall conformations of the three tRNAs (left, E-site in two human 80S ribosomes, P-site in yeast 80S ribosome), the zoom-in views (middle) and the detailed views (right) of the tRNA^{Met} are shown. All tRNAs are shown with density maps indicating their good fit. In the detailed view, the bases are labeled (right). All densities are derived from the cryo-EM density map post-processed by DeepEMhancer.

Supplementary Table 1. Cryo-EM data collection, refinement and validation statistics (part 1)

	55S+5μM Tig	39S+5μM Tig	55S+10μM Tig	39S+10μM Tig	55S+100μM Tig	39S+100μM Tig
EMDB	38632	38633	38634	38635	36836	36837
PDB	8XT0	8XT1	8XT2	8XT3	8K2A	8K2B
**Data collection and processing**						
Magnification	81,000	81,000	81,000	81,000	105,000	105,000
Voltage (kV)	300	300	300	300	300	300
Electron exposure (e–/Å^2)	50	50	50	50	50	50
Defocus range (μm)	-1 to -2.5	-1 to -2.5	-1 to -2.5	-1 to -2.5	-1 to -2.5	-1 to -2.5
Pixel size (Å)	1.064	1.064	1.064	1.064	1.19	1.19
Symmetry imposed	*C1*	*C1*	*C1*	*C1*	*C1*	*C1*
Initial particle images (no.)	1,585,892	1,585,892	1,493,828	1,493,828	1,204,711	1,204,711
Final particle images (no.)	122,982	57,266	83,274	52,961	143,725	12,156
Map resolution (Å)	3.2	3.1	3.3	3.1	2.9	3.4
FSC threshold	0.143	0.143	0.143	0.143	0.143	0.143
Map resolution range (Å)	2.8-12	2.8-11	2.8-11	2.8-11	2.4-16	3-14
**Refinement**						
Initial model used (PDB code)	7A5I	7A5I	7A5I	7A5I	7A5I	7A5I
Model resolution (Å)	3.2	3.1	3.3	3.1	2.9	3.4
FSC threshold	0.5	0.5	0.5	0.5	0.5	0.5
Map sharpening *B* factor (Å^2)	-106	-73	-96	-76	-66	-56
Model composition						
Non-hydrogen atoms	165,243	100,095	165,243	100,095	165,367	102,269
Protein residues	13,759	8,202	13,759	8,202	13,761	8,461
Nucleotides	2,485	1,556	2,485	1,556	2,485	1,556
Ligands	3	1	3	1	4	2
B factors (Å^2)	18.89	54.61	39.58	70.96	57.61	107.01
Protein	19.74	55.66	39.22	76.77	71.00	111.53
Nucleotides	17.02	52.48	40.34	59.24	29.27	97.36
Ligand	31.19	52.67	35.59	58.53	33.14	150.19
R.m.s. deviations						
Bond lengths (Å)	0.0070	0.0041	0.0053	0.0049	0.019	0.012
Bond angles (°)	0.96	0.85	0.89	0.93	1.277	0.808
Validation						
MolProbity score	1.70	1.65	1.70	1.62	2.15	1.95
Clashscore	6.31	5.80	6.37	5.61	11.42	12.06
Poor rotamers (%)	0	0	0.05	0.01	1.52	0.41
Ramachandran plot						
Favored (%)	94.88	95.26	94.87	95.49	93.30	94.82
Allowed (%)	5.06	4.69	5.07	4.45	6.17	5.06
Disallowed (%)	0.06	0.05	0.06	0.06	0.53	0.12

Supplementary Table 2. Cryo-EM data collection, refinement and validation statistics (part 2)

	Human 80S+4µM Tig	Human 80S+100 µM Tig	Human 80S+Tig, E-tRNA, CCDC124	Human 80S+Tig, E-tRNA, eEF2	Human 80S+Tig, E-tRNA, CCDC124 (swiveled)	Human 80S+Tig, E-tRNA, P-tRNA, mRNA	Yeast 80S+Tig, eIF5A, eEF2, Stm1	Yeast 80S+Tig, P-tRNA, Not5
EMDB	39456	39455	36838	38629	38630	38631	36839	36945
PDB	8YOP	8YOO	8K2C	8XSX	8XSY	8XSZ	8K2D	8K82
**Data collection and processing**								
Magnification	130,000	130,000	130,000	130,000	130,000	130,000	130,000	130,000
Voltage (kV)	300	300	300	300	300	300	300	300
Electron exposure (e–/Å^2)	40	40	50	50	50	50	44	44
Defocus range (µm)	-1 to -2.5	-1 to -2.5	-1 to -2.5	-1 to -2.5	-1 to -2.5	-1 to -2.5	-1 to -2.5	-1 to -2.5
Pixel size (Å)	0.727	0.727	0.932	0.932	0.932	0.932	0.847	0.847
Symmetry imposed	*C1*	*C1*	*C1*	*C1*	*C1*	*C1*	*C1*	*C1*
Initial particle images (no.)	591,192	1,377,458	542,730	542,730	542,730	542,730	1,116,932	1,116,932
Final particle images (no.)	98,844	310,531	157,229	88,213	46,089	32,725	105,095	140,917
Map resolution (Å)	2.2	2.0	2.4	2.4	3.0	3.2	3.2	3.0
FSC threshold	0.143	0.143	0.143	0.143	0.143	0.143	0.143	0.143
Map resolution range (Å)	1.8-10	1.8-9	2-10	2-10	2.8-12	2.8-12	2.8-12	2.8-10
**Refinement**								
Initial model used (PDB code)	6Z6L	6Z6L	6Z6L	6Z6M	6Z6L	6Z6L	6Z6J	6TB3
Model resolution (Å)	2.1	1.9	2.4	2.3	3.0	3.1	3.1	3.0
FSC threshold	0.5	0.5	0.5	0.5	0.5	0.5	0.5	0.5
Map sharpening *B* factor (Å^2)	-56	-56	-63	-50	-75	-65	-134	-141
Model composition								
Non-hydrogen atoms	218,499	215,845	224,954	231,068	223,822	222,841	210,169	205,518
Protein residues	11,988	11,779	12,431	13,309	12,431	12,147	12,635	11,675
Nucleotides	5,787	5,665	5,862	5,862	5,856	5,845	5,167	5,351
Ligands	0	6	9	7	8	5	6	7
B factors (Å^2)	25.97	32.49	31.60	49.48	27.72	32.34	24.58	14.62
Protein	16.09	26.40	23.46	50.65	19.70	25.31	24.47	14.43
Nucleotides	33.68	37.23	37.98	48.48	34.06	37.77	24.66	14.76
Other	6.62	33.41	59.29	50.54	37.51	48.18	33.86	17.33
R.m.s. deviations								
Bond lengths (Å)	0.004	0.019	0.007	0.0052	0.0041	0.0037	0.0062	0.020
Bond angles (°)	0.806	0.963	1.038	0.72	0.82	0.80	0.90	1.048
Validation								
MolProbity score	1.90	1.51	1.98	1.88	1.71	1.73	1.83	1.80
Clashscore	4.62	3.44	7.68	9.99	5.54	5.56	6.45	5.78
Poor rotamers (%)	2.49	1.48	1.58	0	0.05	0.02	0.02	0.51
Ramachandran plot								
Favored (%)	94.71	96.34	93.90	94.80	93.81	93.53	92.31	0.25
Allowed (%)	5.18	3.47	5.85	4.99	6.08	6.34	7.44	7.76
Disallowed (%)	0.11	0.19	0.25	0.21	0.11	0.13	0.25	91.99

Supplementary Table 3. Tigecycline molecules in human 80S ribosomes

	Interface on human 18S/28S rRNA or yeast 18S/25S rRNA	80S+4μM Tig	80S+100μM Tig	80S+Tig, E-tRNA, CCDC124	80S+Tig, E-tRNA, eEF2	80S+Tig, E-tRNA, CCDC124 (swiveled)	80S+Tig, E-tRNA, P-tRNA, mRNA	Yeast 80S+Tig, eIF5A, eEF2, Stm1	Yeast 80S+Tig, P-tRNA, Not5
Tig1-2	H68/H75 (28S)	×	■	■	■	■	×	■	■
Tig3	h34 (18S)	×	■	×	×	×	×	×	■
Tig4	ES19 (28S)	×	■	■	■	■	■	×	×
Tig5	H18 (28S)	×	■	■	■	■	■	×	×
Tig6-8	H17 (28S)	×	×	■	■	■	■	×	×
Tig9	E-tRNA	×	×	■	■	×	×	×	×
Tig10	h29 (18S), E-tRNA	×	×	■	×	×	×	×	×
Tig11	H4 (28S)	×	×	×	×	×	×	■	■
Tig12-13	H38 (28S)	×	×	×	×	×	×	■	■

Note: In this table, the presence of the tigecycline molecule is marked by a red color, while its absence is indicated by a black cross.
