# Supplementary material for: Structural basis for differential inhibition of eukaryotic ribosomes by tigecycline
Source: Nat Commun. 2024 Jun 28;15:5481. doi: 10.1038/s41467-024-49797-7 (PMC11213857; doi:10.1038/s41467-024-49797-7)
Supplement: Supplementary file 3 — Reporting Summary [file 41467_2024_49797_MOESM3_ESM.pdf]

nature portfolio

Corresponding author(s): Dr. Jingdong Cheng
Last updated by author(s): May 31, 2024

Reporting Summary

Nature Portfolio wishes to improve the reproducibility of the work that we publish. This form provides structure for consistency and transparency in reporting. For further information on Nature Portfolio policies, see our Editorial Policies and the Editorial Policy Checklist.

Statistics

For all statistical analyses, confirm that the following items are present in the figure legend, table legend, main text, or Methods section.

n/a	Confirmed	
☐	☒	The exact sample size (*n*) for each experimental group/condition, given as a discrete number and unit of measurement
☐	☒	A statement on whether measurements were taken from distinct samples or whether the same sample was measured repeatedly
☒	☐	The statistical test(s) used AND whether they are one- or two-sided<br>*Only common tests should be described solely by name; describe more complex techniques in the Methods section.*
☒	☐	A description of all covariates tested
☒	☐	A description of any assumptions or corrections, such as tests of normality and adjustment for multiple comparisons
☐	☒	A full description of the statistical parameters including central tendency (e.g. means) or other basic estimates (e.g. regression coefficient) AND variation (e.g. standard deviation) or associated estimates of uncertainty (e.g. confidence intervals)
☒	☐	For null hypothesis testing, the test statistic (e.g. *F*, *t*, *r*) with confidence intervals, effect sizes, degrees of freedom and *P* value noted<br>*Give P values as exact values whenever suitable.*
☒	☐	For Bayesian analysis, information on the choice of priors and Markov chain Monte Carlo settings
☒	☐	For hierarchical and complex designs, identification of the appropriate level for tests and full reporting of outcomes
☒	☐	Estimates of effect sizes (e.g. Cohen's *d*, Pearson's *r*), indicating how they were calculated

Our web collection on statistics for biologists contains articles on many of the points above.

Software and code

Policy information about availability of computer code

Data collection	Cryo-EM data were collected using EPU v2 software.
Data analysis	For cryo-EM data: 1) Movies were aligned using MotionCor2. 2) CTF parameters and resolutions were estimated using CTFFIND4 and GCTF. 3) Particle picking was performed in Gautomatch v0.56. 4) Data were processed in Relion 3.1 and cryoSPARC v3.2. 5) Model building was done in COOT 0.9. 6) Models were refined using Phenix 1.19 and evaluated using MolProbity. 7) Maps were filtered using Relion 3.1 and DeepEMhancer 0.3.<br>All figures showing cryo-EM maps and the molecular model were generated in ChimreaX v1.6.<br>All plots were created using GraphPad Prism 8.<br>Gray scale calculation of protein bands in Figure 1 was performed using Image Lab 6.1(Bio-Rad) and ImageJ1.

For manuscripts utilizing custom algorithms or software that are central to the research but not yet described in published literature, software must be made available to editors and reviewers. We strongly encourage code deposition in a community repository (e.g. GitHub). See the Nature Portfolio guidelines for submitting code & software for further information.

Data

Policy information about availability of data

All manuscripts must include a data availability statement. This statement should provide the following information, where applicable:

- Accession codes, unique identifiers, or web links for publicly available datasets
- A description of any restrictions on data availability
- For clinical datasets or third party data, please ensure that the statement adheres to our policy

All cryo-EM maps and molecular models generated in this study have been deposited in the Electron Microscopy Data Bank (EMDB) and in the Protein Data Bank (PDB) with accession codes: EMD-36836 [https://www.ebi.ac.uk/emdb/EMD-36836] and 8K2A [http://doi.org/10.2210/pdb8K2A/pdb] for the 55S mitoribosome + 100 µM tigecycline; EMD-36837 [https://www.ebi.ac.uk/emdb/EMD-36837] and 8K2B [http://doi.org/10.2210/pdb8K2B/pdb] for the 39S mitoribosome + 100 µM tigecycline; EMD-36838 [https://www.ebi.ac.uk/emdb/EMD-36838] and 8K2C [http://doi.org/10.2210/pdb8K2C/pdb] for the human 80S ribosome with tigecycline, E-tRNA and CCDC124; EMD-36839 [https://www.ebi.ac.uk/emdb/EMD-36839] and 8K2D [http://doi.org/10.2210/pdb8K2D/pdb] for the yeast 80S ribosome with tigecycline, eEF2, Stm1 and eIF5A; EMD-36945 [https://www.ebi.ac.uk/emdb/EMD-36945] and 8K82 [http://doi.org/10.2210/pdb8K82/pdb] for the yeast 80S ribosome with tigecycline, Not5 and P-tRNA; EMD-38632 [https://www.ebi.ac.uk/emdb/EMD-38632] and 8XT0 [http://doi.org/10.2210/pdb8XT0/pdb] for the 55S mitoribosome + 5 µM tigecycline; EMD-38633 [https://www.ebi.ac.uk/emdb/EMD-38633] and 8XT1 [http://doi.org/10.2210/pdb8XT1/pdb] for the 39S mitoribosome + 5 µM tigecycline; EMD-38634 [https://www.ebi.ac.uk/emdb/EMD-38634] and 8XT2 [http://doi.org/10.2210/pdb8XT2/pdb] for the 55S mitoribosome + 10 µM tigecycline; EMD-38635 [https://www.ebi.ac.uk/emdb/EMD-38635] and 8XT3 [http://doi.org/10.2210/pdb8XT3/pdb] for the 39S mitoribosome + 10 µM tigecycline; EMD-38629 [https://www.ebi.ac.uk/emdb/EMD-38629] and 8XSX [http://doi.org/10.2210/pdb8XSX/pdb] for the human 80S ribosome with tigecycline, E-tRNA, SERBP1 and eEF2; EMD-38630 [https://www.ebi.ac.uk/emdb/EMD-38630] and 8XSY [http://doi.org/10.2210/pdb8XSY/pdb] for the human 80S ribosome with tigecycline, E-tRNA and CCDC124 (40S head swiveled); EMD-38631 [https://www.ebi.ac.uk/emdb/EMD-38631] and 8XSZ [http://doi.org/10.2210/pdb8XSZ/pdb] for the human 80S ribosome with tigecycline, E-tRNA, P-tRNA and mRNA; EMD-39455 [https://www.ebi.ac.uk/emdb/EMD-39455] and 8YOO [http://doi.org/10.2210/pdb8YOO/pdb] for the human empty 80S ribosome with 100 µM tigecycline; EMD-39456 [https://www.ebi.ac.uk/emdb/EMD-39456] and 8YOP [http://doi.org/10.2210/pdb8YOP/pdb] for the human empty 80S ribosome with 4 µM tigecycline. Source data are provided with this paper.

Research involving human participants, their data, or biological material

Policy information about studies with human participants or human data. See also policy information about sex, gender (identity/presentation), and sexual orientation and race, ethnicity and racism.

Reporting on sex and gender	N/A
Reporting on race, ethnicity, or other socially relevant groupings	N/A
Population characteristics	N/A
Recruitment	N/A
Ethics oversight	N/A

Note that full information on the approval of the study protocol must also be provided in the manuscript.

Field-specific reporting

Please select the one below that is the best fit for your research. If you are not sure, read the appropriate sections before making your selection.

[x] Life sciences   [ ] Behavioural & social sciences   [ ] Ecological, evolutionary & environmental sciences

For a reference copy of the document with all sections, see nature.com/documents/nr-reporting-summary-flat.pdf

Life sciences study design

All studies must disclose on these points even when the disclosure is negative.

Sample size	The sample size for the cryo-EM data was estimated manually to ensure enough particles in each subclass to obtain good resolution for the cryo-EM reconstructions. The sample sizes for the tigecycline inhibition assays were determined according to the previous studies from our laboratories and similar published work in the literature.
Data exclusions	No data was excluded from the experiments
Replication	L-AHA labeling on nascent proteins (Cyto and Mito), in vitro translation, and CCK-8 cell growth assay were performed at least in triplicate. All the replication were successful.
Randomization	This is not relevant since particle distribution is assigned using maximum likelihood approaches in Relion software.
Blinding	The investigators were blinded to group allocation during data collection and analysis.

Reporting for specific materials, systems and methods

We require information from authors about some types of materials, experimental systems and methods used in many studies. Here, indicate whether each material, system or method listed is relevant to your study. If you are not sure if a list item applies to your research, read the appropriate section before selecting a response.

Materials & experimental systems

n/a	Involved in the study
☒	☐ Antibodies
☐	☒ Eukaryotic cell lines
☒	☐ Palaeontology and archaeology
☒	☐ Animals and other organisms
☒	☐ Clinical data
☒	☐ Dual use research of concern
☒	☐ Plants

Methods

n/a	Involved in the study
☒	☐ ChIP-seq
☒	☐ Flow cytometry
☒	☐ MRI-based neuroimaging

Eukaryotic cell lines

Policy information about cell lines and Sex and Gender in Research

Cell line source(s)	HEK293T or SK-Hep1 was kindly gifted by Prof. Dan Ye, in Fudan University, China.<br>Expi293 cells were purchased from Thermo Fisher (A14527).<br>FreeStyle 293-F cells were purchased from Thermo Fisher (R79007).
Authentication	The cell lines HEK293T and SK-Hep1 were not authenticated by ourself. Expi293 and FreeStyle 293-F cells were authenticated by Thermo Fisher.
Mycoplasma contamination	The cell lines were not tested for Mycoplasma contamination by ourself.
Commonly misidentified lines (See ICLAC register)	No commonly misidentified cell lines were used in the study.

Plants

Seed stocks	*Report on the source of all seed stocks or other plant material used. If applicable, state the seed stock centre and catalogue number. If plant specimens were collected from the field, describe the collection location, date and sampling procedures.*
Novel plant genotypes	*Describe the methods by which all novel plant genotypes were produced. This includes those generated by transgenic approaches, gene editing, chemical/radiation-based mutagenesis and hybridization. For transgenic lines, describe the transformation method, the number of independent lines analyzed and the generation upon which experiments were performed. For gene-edited lines, describe the editor used, the endogenous sequence targeted for editing, the targeting guide RNA sequence (if applicable) and how the editor was applied.*
Authentication	*Describe any authentication procedures for each seed stock used or novel genotype generated. Describe any experiments used to assess the effect of a mutation and, where applicable, how potential secondary effects (e.g. second site T-DNA insertions, mosiacism, off-target gene editing) were examined.*
